# Supplementary material for: Differential CpG DNA methylation in peripheral naïve CD4+ T-cells in early rheumatoid arthritis patients
Source: Clin Epigenetics. 2020 Apr 7;12:54. doi: 10.1186/s13148-020-00837-1 (PMC7137446; doi:10.1186/s13148-020-00837-1)
Supplement: Supplementary file 3 — Additional file 3: Supplementary methods and results. [file 13148_2020_837_MOESM3_ESM.docx]

**Supplement methodology**

*ELISA*

Peripheral blood (9 ml clotting tube) was collected at the inclusion visit. After centrifugation, serum was stored at -80⁰C until analysis. Three commercial enzyme-linked immunosorbent assays (ELISA) kits were used to measure cytokines: RANKL (BioVENDOR, CZR), IL-21 and IL-34 (Biolegend, UK). ELISA was performed according to manufacturer’s instructions.

*Bisulfite sequencing of the TNF-alpha promoter region*

CD4 T-cells were isolated (CD4+ T cell enrichment kit STEMCELL, UK) with ~ 97% purity. DNA was extracted (QIAamp DNA blood mini kit, QIAGEN, UK), and bisulfite converted using the EZ DNA methylation-Gold^TM^ (Zymo Research, USA) kit. To amplify a small region in the promoter of the TNF gene, a polymerase chain reaction (PCR) was performed (containing HotStarTaq enzyme, dNTPs, buffer, QIAGEN, UK), 500 nM of forward and reverse primers (F5’-GAGTGTGAGGGGTATTTTTGATGTT-3’), (R5’-CTCTCCCTCTTAACTAATCCTCTACTA TCC-3’), 1mM MgCl_2_ and 2 µL of converted DNA. PCR conditions were 1 cycle of denaturation (10 min at 95°C), followed by 40 cycles of amplification (94°C for 10s, 59°C for 20s and 72°C for 45s), and a final extension (72 °C for 10 min). The PCR product (2.5 µL) was add to 1 µL of ExoProStar enzyme (Illustra^TM^ ExoProStar^TM^, GE Healthcare, UK) and placed in the thermocycler for 1 cycle of 15 min at 37°C, followed by 15 min at 80°C. The PCR product was finally diluted with 3.5 µL of nuclease-free water before use in the sequencing step. BigDye Terminator (V3.1 Cycle sequencing kits,) was used for sequencing: 0.25 µL of “Ready Reaction Mix”, 3.5 µl of ABI sequencing buffer and 0.16 µM primer (forward and reverse reaction done separately) were add to 1ul of PCR product in 10 µl total volume. The mix was placed on a thermocycler for 28 cycles (96°C for 10 sec, 50°C for 5 sec, and 60°C for 4 min). The samples were then precipitated with ethanol and the DNA pellets dried for 1 minute at 95°C. Pellets were re-suspended in 20 µL of HiDi formamide and sequenced on the 3130xl Genetic Analyzer (ABI Prism, USA). CpGmethylated Hela Genomic DNA was used as reference of fully methylated DNA.

*Analysis of publicly available gene expression data*

Gene expression data were obtained from ArrayExpress (E-GEOD-20098, E-GEOD-26163)(1, 2). Sample were CD4+T-cells, purified from peripheral blood of 47 early, drug naïve RA patients and 16 HC. These datasets were both generated using Illumina HumanWG-6 v3.0 expression beadchips and BeadStudio version 3.3.7 software (Illumina, San Diego, California, USA). Pre-processing of raw data was performed using the *Lumi* package(3, 4) for data input, quality assessment, and normalisation (robust spline) on log_2_-transfromed data. The *normalizeBetweenArrays* function within the Limma Package was used to achieve consistency between the two arrays (Ritchie et al, 2007) (supplementary Figure S7A). The data was then filtered for probe signal intensity: probes which had a p value ≤0.05 in at least 10% of samples were retained and aggregated to genes using Limma’s avereps function. Linear models made using Limma version 3.34.9 were used to assess differential gene expression(5, 6). The empirical Bayes method was employed to moderate the standard errors of the estimated fold-changes(6), and the *arrayWeights* function(7) measured how well the expression values followed the linear model. Correction for multiple testing was done by the Benjamini-Hochberg method; taking an adjusted p value of ≤0.05(8).

*Subset phenotyping by flow cytometry*

Flow cytometry was performed using standard cell surface staining protocol using fresh EDTA blood, following red cell lysis. Naïve CD4+T-cells were gated based on the expression of CD3/CD4/CD45RA/CD45RO (as described above). The expression of CD4, IL-6R (CD126 clone M5, BD), IL-2R (CD25 clone 2A3, BD), CXCR4 (CD184 clone 12G5, BD), IL-7R (CD127 clone R34.34, Beckman Coulter)) was measured on naïve CD4+T-cells using Mean Fluorescence Intensity (MFI). Expression of CD62L (clone 145/15, Miltenyi) was either positive or negative and the percentage of CD3+/CD4+/CD45RA+/CD62L- cells was recorded.

*Statistical Analysis*

Sample numbers are reported in the figure legends. Data were displayed as box-plot (ELISA or MFI). Non-parametric Mann-Whitney U-test was performed on data comparing HC and RA. Statistical analysis was performed in SPSS V24.

*Antibody clone details used for flow cytometry*

Antibody Company

| Mouse Anti-Human CD4 Monoclonal Antibody (Clone SK3) | BD Biosciences |
| --- | --- |
| Mouse Anti-Human CD3 Monoclonal Antibody (Clone UCHT1) | BD Biosciences |
| Mouse Anti-Human CD45RA Monoclonal antibody (Clone F8-11-13) | AbD Serotec |
| Mouse Anti-Human CD45RB Monoclonal antibody (Clone MEM-55) | AbD Serotec |
| Mouse Anti-Human CD45RO Monoclonal antibody (Clone UCHL1) | BD Biosciences |
| Mouse Anti-Human CD14 Monoclonal Antibody (Clone M5E2) | BD Biosciences |
| Mouse Anti-Human CD25 Monoclonal Antibody (Clone2A3) | BD Biosciences |
| Mouse Anti-Human CD126 (Clone M5) | BD Biosciences |
| Mouse Anti-Human CD127 Monoclonal Antibody (Clone HIL-7R-M21) | BD Biosciences |
| Mouse Anti-Human CD62L Monoclonal Antibody (Clone 145/15)  Mouse Anti-Human CD184 Monoclonal Andibody (Clone 12G5) | Miltenyi Biotec  BD Biosciences |

**Supplement Table S1.**  Demographic and clinical data

| **Cohort-1 : DNA methylation array** | **HC (n=6)** | **RA (n=10)** |  |
| --- | --- | --- | --- |
| age (years)* | 42 (38-47) | 50 (40-74) |  |
| M/F | 3/3 | 7/3 |  |
| ACPA (Pos/Neg) | na | 6/4 |  |
| Duration (months)* | na | 13 (3-24) |  |
| TJC | na | 10 (3-16) |  |
| SJC | na | 3 (3-11) |  |
| CRP | na | 20 (10-40) |  |
| **Cohort 2 : bisulfite sequencing** | **HC (n=7)** | **RA (n=9)** | |
| age (years)* | 55 (48-63) | 46 (31-65) |  |
| M/F | 1/6 | 3/6 |  |
| ACPA (Pos/Neg) | na | 7/2 |  |
| Duration (months)* | na | 15 (5-24) |  |
| TJC | na | 5 (3-18) |  |
| SJC | na | 3 (1-6) |  |
| CRP | na | 10 (<5-83) |  |
| **Cohort 3 : ELISA** | **HC (n=10)** | **RA (n=20)** | |
| age (years)* | 51 (40-63) | 60 (41-75) |  |
| M/F | 4/6 | 7/17 |  |
| ACPA (Pos/Neg) | na | 14/6 |  |
| Duration (months)* | na | 4 (1-24) |  |
| TJC | na | 12 (5-22) |  |
| SJC | na | 11 (0-22) |  |
| CRP | na | 20 (<5-60) |  |
| **Cohort 4 : Flow cytometry** | **HC (n=10)** | **RA (n=35)** | |
| age (years)* | 44 (26-59) | 54 (26-76) | |
| M/F | 5/5 | 7/13 | |
| ACPA (Pos/Neg) | na | 15/5 | |
| Duration (months)* | na | 4 (1.5-12) | |
| TJC | na | 9 (0-28) | |
| SJC | na | 5 (0-20) | |
| CRP | na | 6 (<5-151) | |

Δβ-values analysis (List 2,3)

Data Analysis Work Flow

Methylation dataset (3 cell subsets)

Quality control and data pre-processing

Preliminary exploration:

MDS, t-test, Manhattan plot, Heatmap

Explore data with a prioritisation scoring system (Table 1)

List of DM-CpGs using

Validation

DMRcates R package (List 1)

TNFgene Bisulfite sequencing

STRING Network analysis

In silico Gene expression

Cell surface marker analysis by flow cytometry

**Supplement Figure S1.** DNA methylation data analysis workflow.


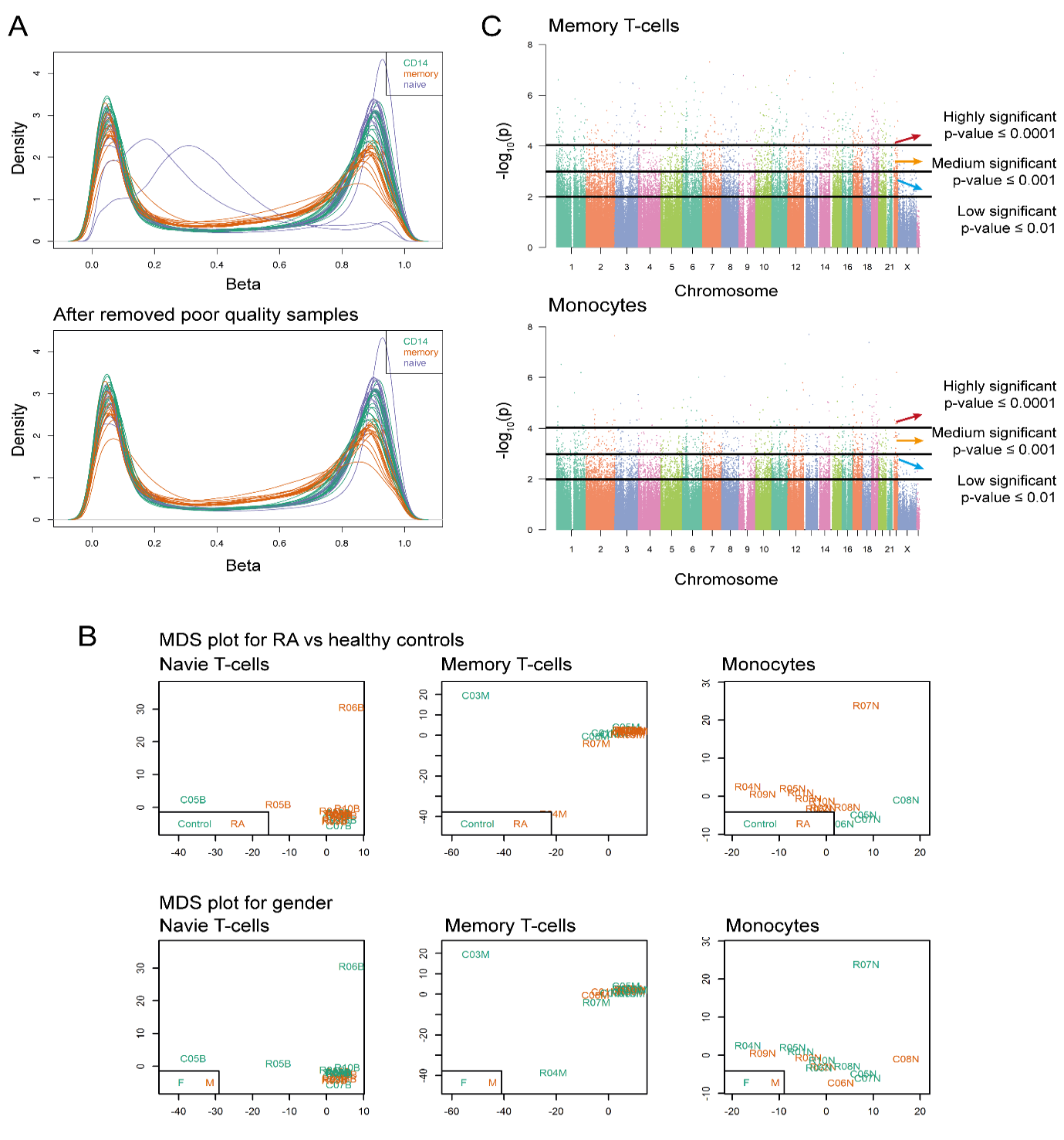


**Supplement Figure S2.** **Quality control, data pre-processing and data mining**

A) Beta value histograms for the 48 samples: 2 failed quality analysis due to too low amount of DNA. 0-unmetlylated and 1-methylated with intermediate values when 2 population of cells are present. 2 samples showing deviation were excluded. B) Preliminary exploration using MDS for gender and RA versus HC. MDS did not segregate samples for gender bias, after exclusion of Chromosome X and Y data) or for RA (n=10) versus HC (n=6). C) Manhattan plot for -Log_10_(p-values) against position on chromosome of individual CpG for memory T-cells and Monocytes.

**
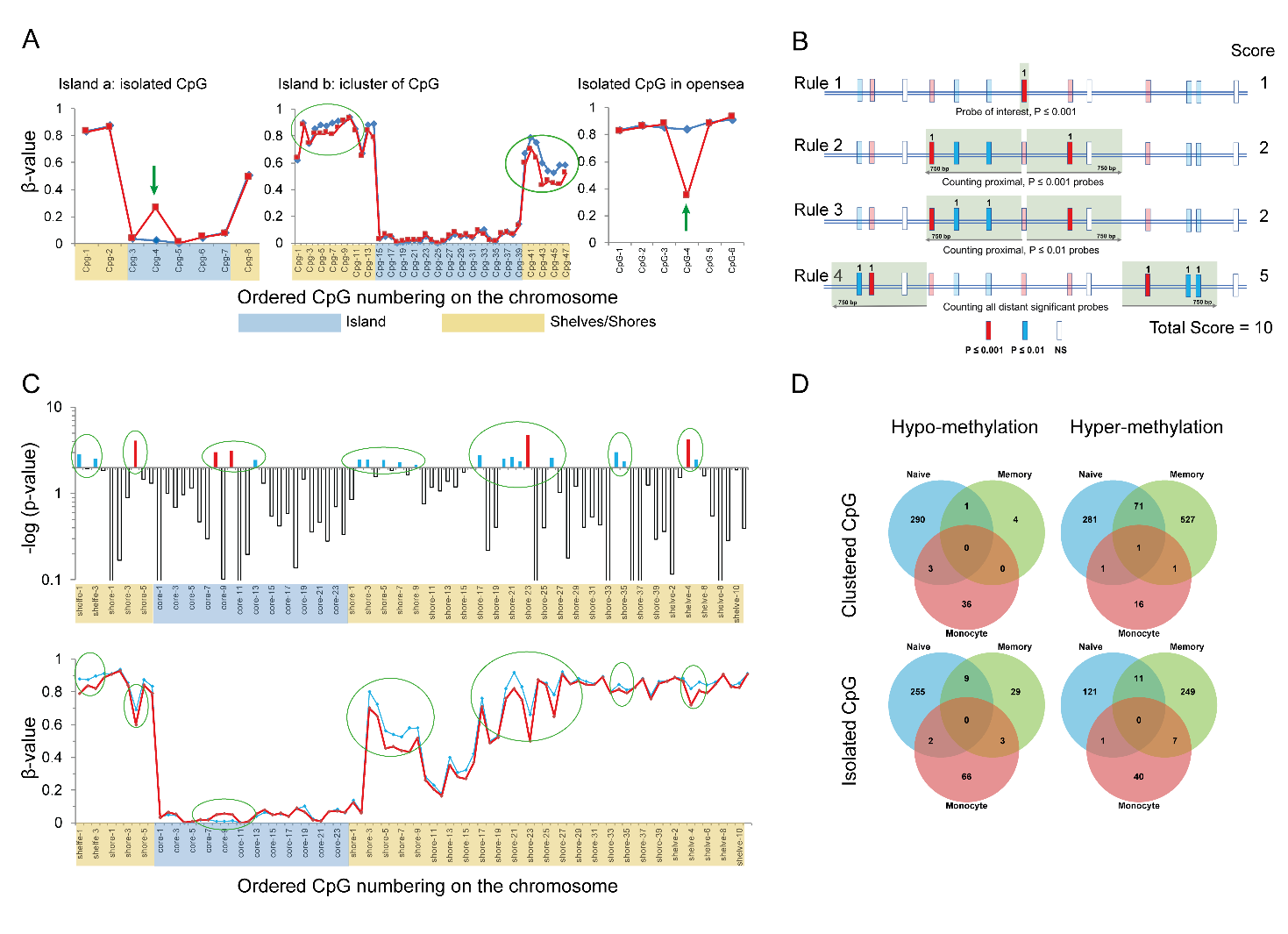
**

**Supplement Figure S3.**

A). **Typical patterns of the differential methylation**. In example a) the most significant DM-CpG observed (CpG06292898; p=1.06^e-17^), showed very clear differential levels of methylation between HC and RA patients, however this was associated with a single CpG in a 321 bp long island, where no other difference was observed for any of the other 7 probes. Consensus binding sites for the transcription factors SP1, TFAP2c, PAX-5 or E2F1 are localised nearby this CpG although not directly overlapping; they could nonetheless be influenced by it. *In CpG island-b*, 16/47 probes associated with the island, showed medium/high significant DM p-values (0.0003<p<0.001) and were clearly clustered in regions covering about 900 bp on the left of the core-island (shore/shelve) and 150 bp on its right. The close proximity of these alterations suggests they could locally have a cumulative effect on the chromatin and alter gene expression. *In example c*, an isolated, highly significant DM-CpG was observed (p=5.78^e-13^) far from any recorded island/gene or from the next CpG interrogated by a probe (over 10,000 bp). An effect is difficult to understand (especially when not related to any gene) however this could be a CpG located in an enhancer region, which are not targeted/annotated in this array.

B**). Rule design**. Using only CpG-probes that had a high significant p-value (p≤0.0001), rule-1 score 1 point vs. none. Probes of high/medium significance in a proximal region (+/-750bp, about 3 nucleosomes) were counted in rule-2 (1 point for each probe). Probes of low significance were separately counted in the same proximal region as rule-3 (1 point for each probe). Finally, rule-4 counted probes at all levels of significance (p≤0.01) in a distal region covering a further +/-750pb as (1 point for each probe).

C**) Highest score example**. the top panel represents the log(p-value) of ordered CpG for the B3GALT4 gene, displaying 5 highly significant CpG (in red) then lower significant CpG (in blue) and non-significant CpG (in white, inverted). Clusters of significant CpG are depicted by bubbles. Out of the 5 initial selecting probes (rule 1), the best one yielded a score of 13, adding up significant CpGs spread in the nearby regions. On the bottom panel, a plot of the median β-values for each CpG shows difference between HC (blue) and RA (red).

D**) Venn diagram** overlap between cell subset for clustered and isolated DM-CpGs, hypo or hyper-methylated.


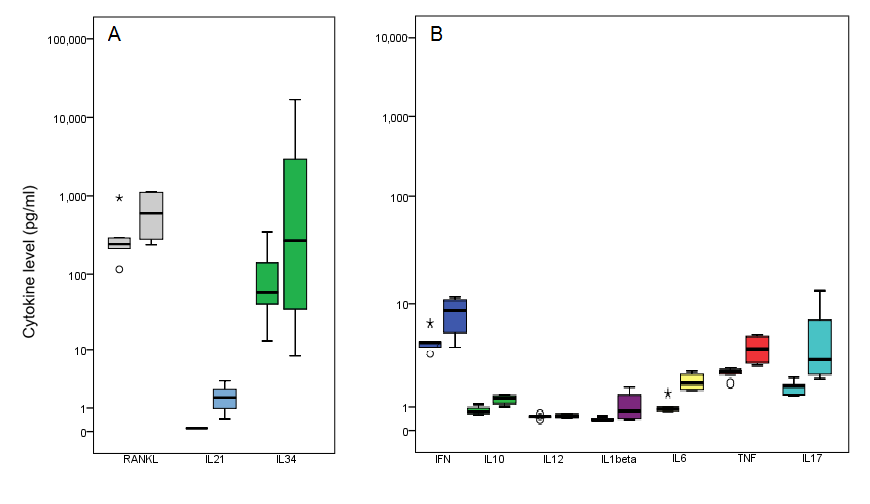


**Supplement Figure S4.**  Levels of expression of several cytokine in HC (left box plot in each pair) versus early RA (right box plot) serum samples using ELISA. Circles are outliers and stars extremes of distribution. The samples were collected from HC (n=10) and early RA (n=20). All cytokines were significantly over expressed in RA (Mann-Whitney U-test , p≤0.001). A) Novel data. B) Data recapitulated from previous studies.


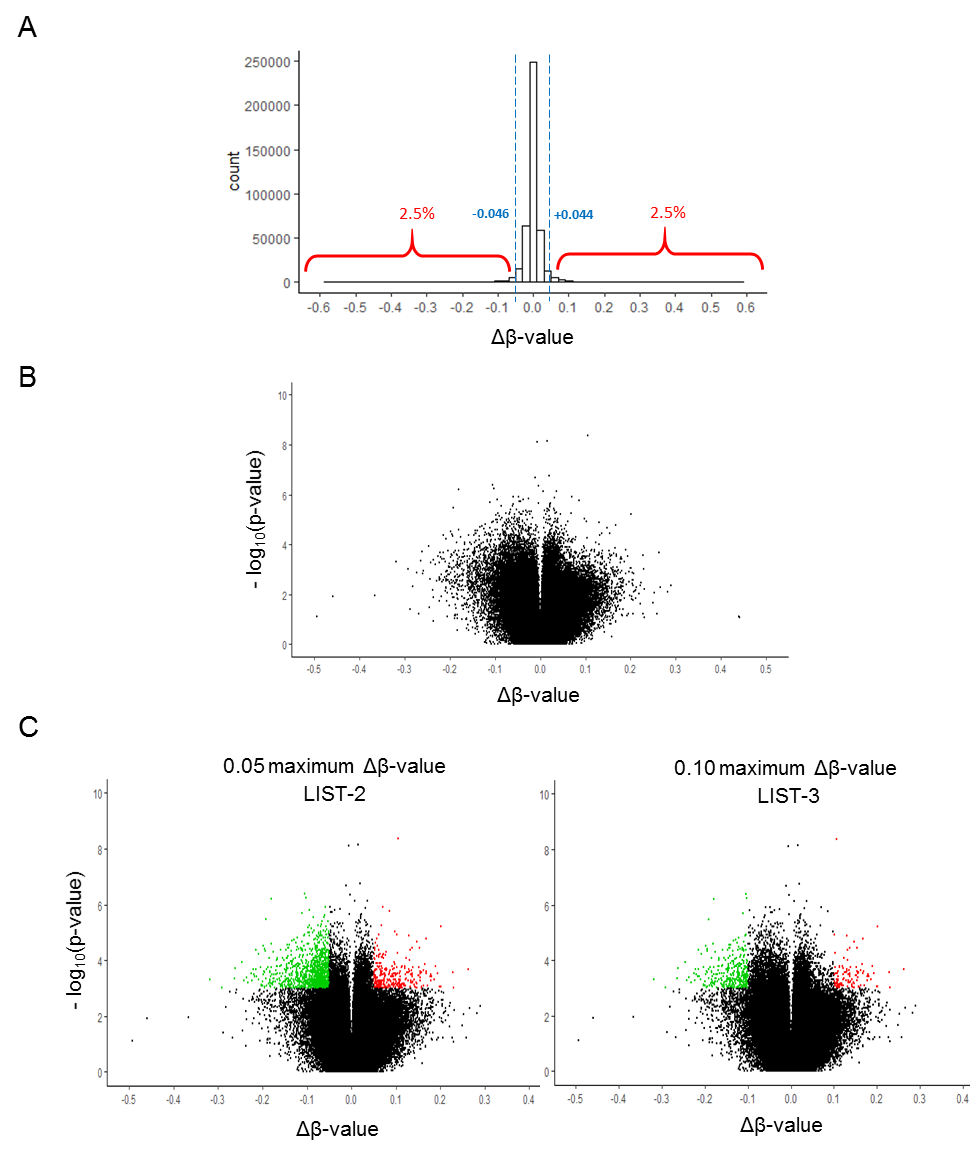


**Supplement Figure S5. Adjustment of Δβ-values cut-off**

1. Distribution of Δβ−values for each CpGs on the array between RA and HC showing a majority of values with a small delta and a significant fraction of Δβ-values spread over a large range. Using 2.5% of all values on each side, cut-off for significance were established at under -0.046 and over +0.044.
2. Volcano plot data for all CpG on the array showing more hypomethylation (left side) in RA than hypermethylation (right).
3. Significant DM-CpG based on 0.05 and 0.1 cut-off for maximum Δβ-values and a p ≤ 0.001 used to generate LIST-2 and 3.


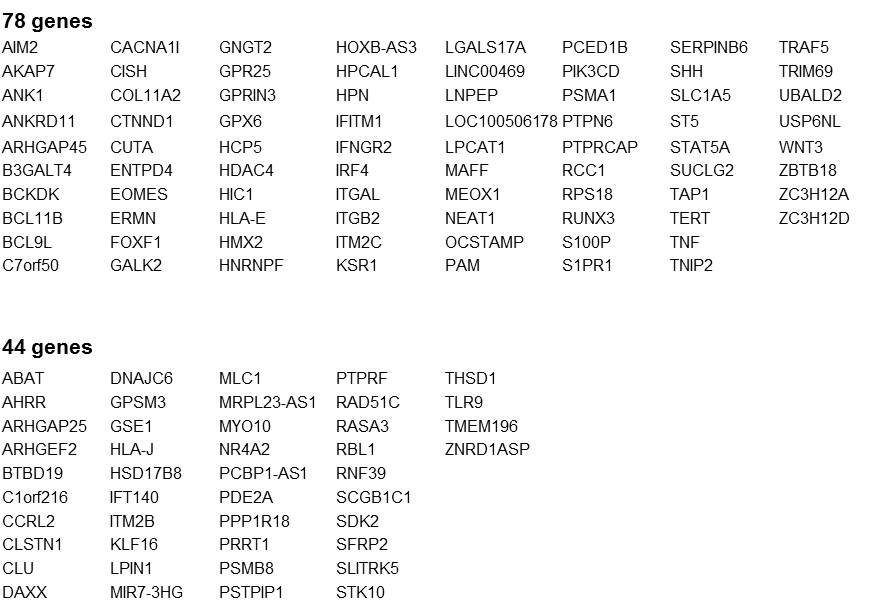


277

LIST-1

LIST-2

LIST-3

78

44

818

81

**Supplement Figure S6**. Overlap between results obtained with 3 DNA methylation data analysis strategies. Data were analysed using DMRcate package (LIST-1) and using Δβ-values with a cut-off at 0.05 (LIST-2) and at 0.10 (LIST-3). Overlap between results are presented as Venn diagrams. The list of overlapping gene are showed in a table.

**
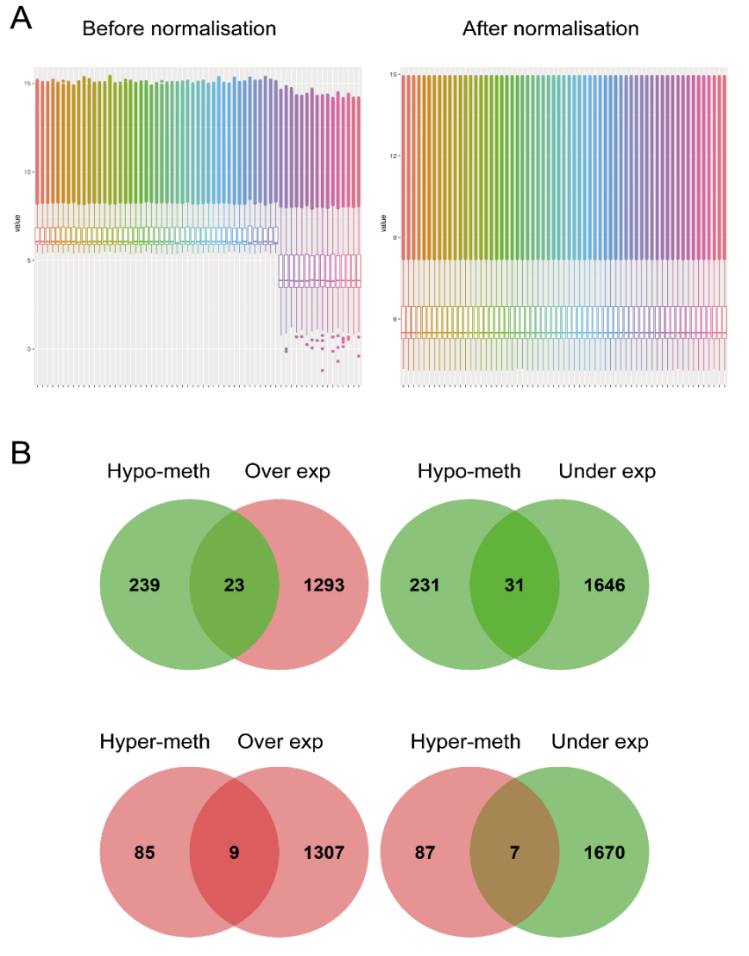
**

| **23 Hypo-Over** | | | **31 Hypo-Under** | | | **9 Hyper-Over** | **7 Hyper-Under** |
| --- | --- | --- | --- | --- | --- | --- | --- |
| CTLA4 | MAF | TAB2 | B3GALT4 | GRK6 | SH2B3 | AFF1 | ATP6V1B2 |
| FAM46C | MICB | UBALD2 | BCKDK | IFITM3 | SLC19A1 | ARIH1 | EOMES |
| FBXO32 | MTFP1 | UBE2H | BCL9L | IL10RA | SLC1A5 | CD2AP | HVCN1 |
| GPRIN3 | NCK2 |  | CISH | IL16 | SMAD3 | HMGB1 | IRF8 |
| HELB | PCED1B |  | CORO1A | ITM2C | STAT5A | LINC00938 | KCTD13 |
| HLA-F-AS1 | PCGF5 |  | DENND2D | NCOR2 | TMC6 | PELI1 | SPIB |
| IFITM1 | PSMA1 |  | ENTPD4 | PIK3CD | TNFRSF8 | PHTF2 | TTYH3 |
| IGFL2 | PSTPIP2 |  | FBRSL1 | PISD | TRIB1 | PRDM1 |  |
| IL2RA | S1PR1 |  | FBXL16 | PTPN6 | UBR4 | TMEM30B |  |
| KCNA3 | SUCLG2 |  | GIMAP4 | RIN3 | ZC3H12A |  |  |
|  |  |  |  |  | ZDHHC18 |  |  |

**Supplement Figure S7. Gene expression analysis.**

A) Quality control and data pre-processing from 2 datasets for gene expression in CD4+T-cell. B) Venn diagram and table of overlapping genes between DEG (fold change expression ≥1.5, with adjusted p-value ≤ 0.05) and DM-genes from LIST-3.

*STRING network analysis of memory T-cells and monocytes DM-genes.*

A similar STRING analysis was performed on memory and monocyte. Hypermethylation was mainly observed in memory T-cells, suggesting a potential global gene silencing effect (by analogy to the cancer field). The STRING analysis of the isolated-DM-CpGs and DM-CpG-clusters (n=502), suggested a single major node, centred on EP300/SKG1 (Figure S6A). This node then linked to several other genes, histone modification enzymes (Hist3H2A), transcription factors (HES5, PAX6, FoxO3) and signalling protein (TRAF6). EP300 encodes the histone acetyltransferase p300 that regulates transcription of genes playing an essential role in cell growth and/or differentiation, notably preventing tumor growth. EP300 contains a domain that recognizes acetylated lysine residues bromodomain that is known to be involved in IL6 signaling(9) and as a co-activator of hypoxia-inducible factor 1 alpha (HIF1A) resulting in VEGF induction. IL6 signalling therefore appears also central to the memory networks via EP300. Serine/threonine-protein kinase (SGK1) regulates ion transport and is under the control of stimuli including insulin (as seen here with the insulin receptor gene INSR), growth factors and glucocorticoids(10). It has been shown to contribute to several pathways including inflammation, cell proliferation and apoptosis(11).

A similar analysis run for monocyte specific DM-genes (n=187) revealed no particular dominant node (Figure S6B). Although IL6R/IL6 were not themselves DM in monocytes, EP300 remained central to the network generated for this subset.

**
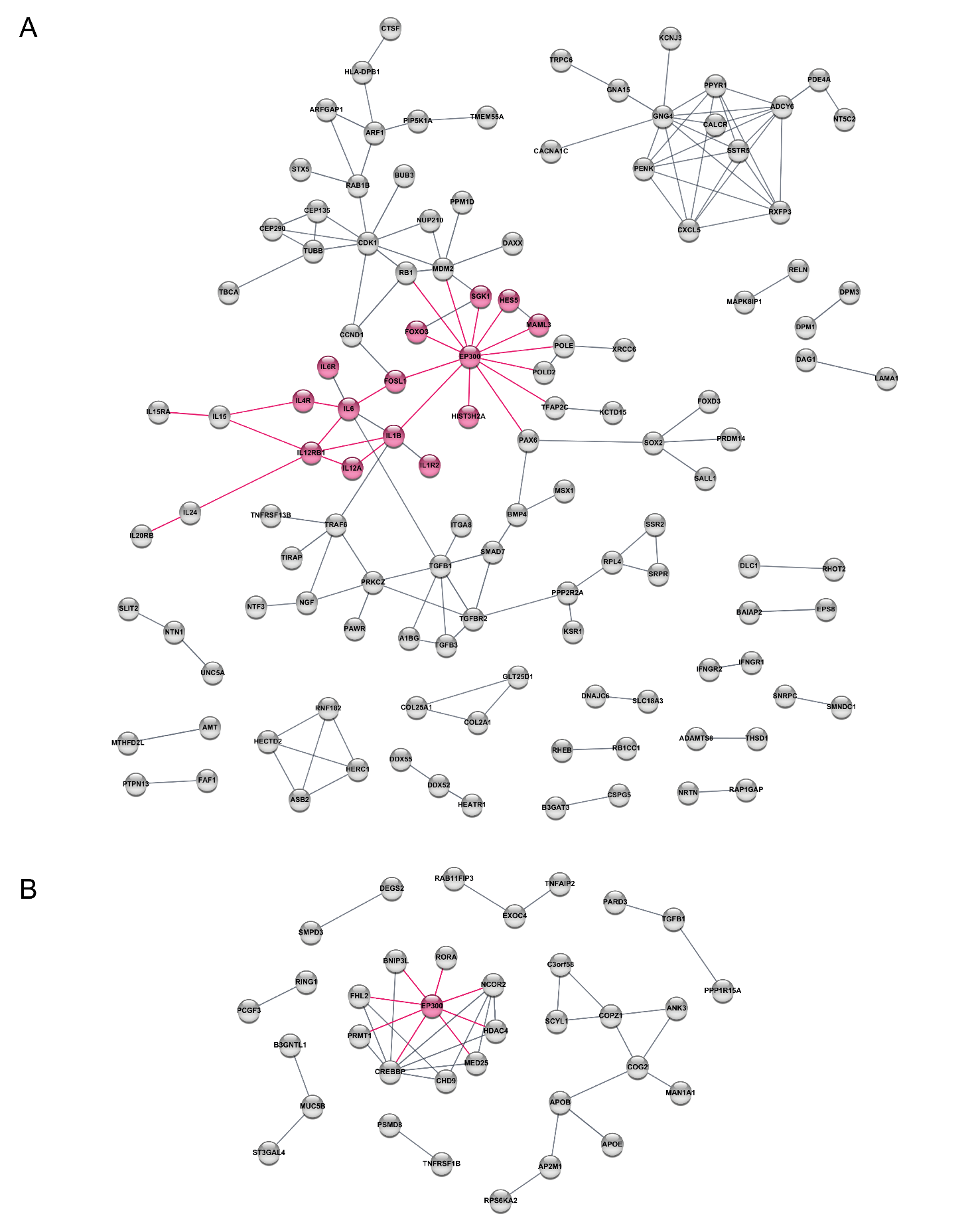
**

**Supplement Figure S8**. STRING network of functional relationships between DM-genes in A) memory T-cells and B) monocytes.

**
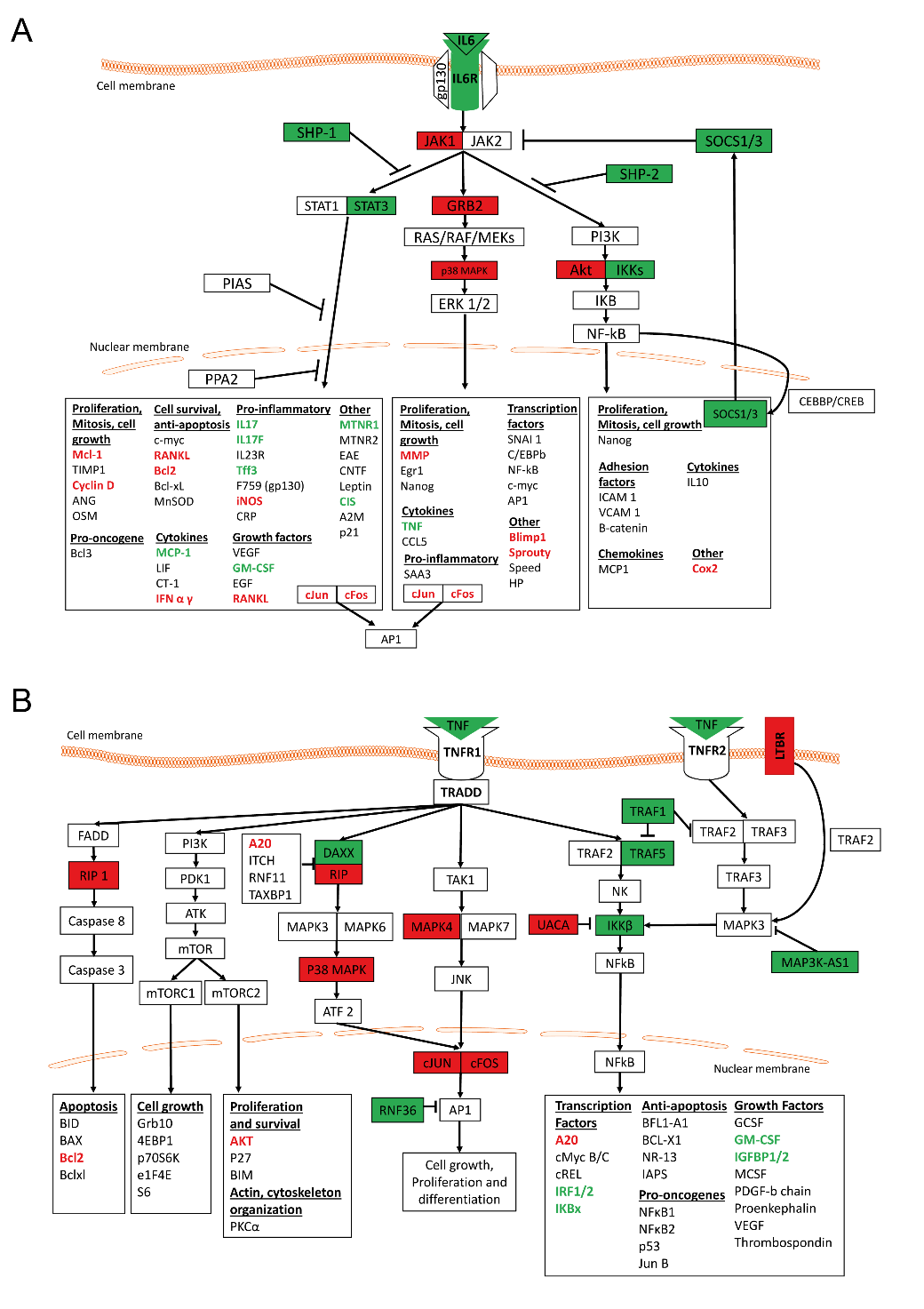

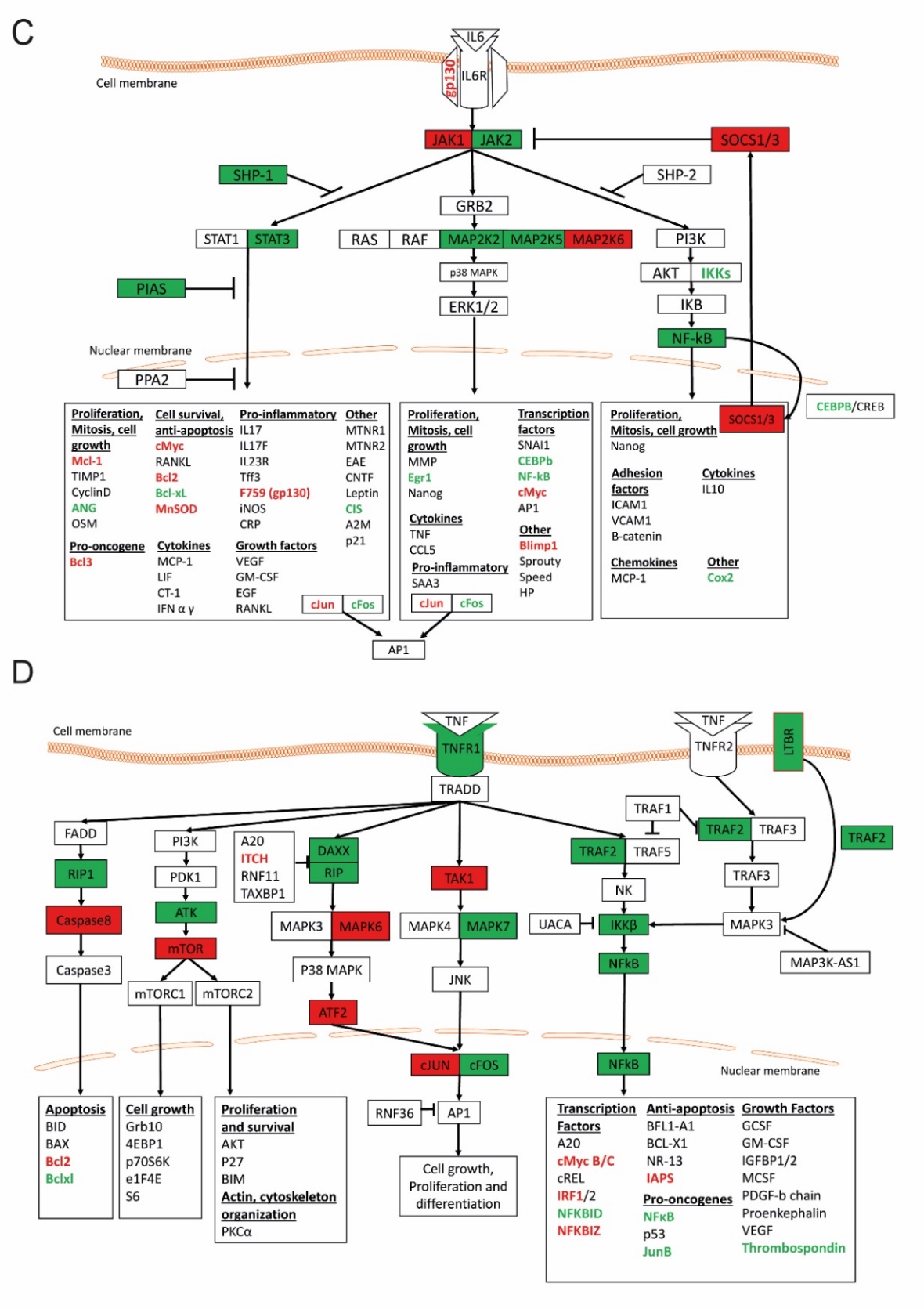
**

**Supplement Figure S9.**  **Reconstitution of the signalling cascade for** **IL6 and TNF-alpha.**

All components of the signalling cascades were listed (from several data knowledge sources) and assembled in a signalling cascade schematic.

A-B) When matched, hypomethylated DM-gene symbols are represented in green; hypermethylated DM-gene in red.

C-D) When matched with DEG, under expressed gene are represented in green and over expressed gene in red.

**References**

1. Pratt AG, Swan DC, Richardson S, Wilson G, Hilkens CM, Young DA, et al. A CD4 T cell gene signature for early rheumatoid arthritis implicates interleukin 6-mediated STAT3 signalling, particularly in anti-citrullinated peptide antibody-negative disease. Annals of the rheumatic diseases. 2012.

2. Gruden K, Hren M, Herman A, Blejec A, Albrecht T, Selbig J, et al. A "crossomics" study analysing variability of different components in peripheral blood of healthy caucasoid individuals. Plos One. 2012;7(1):e28761.

3. Lin SM, Du P, Huber W, Kibbe WA. Model-based variance-stabilizing transformation for Illumina microarray data. Nucleic Acids Res. 2008;36(2):e11.

4. Du P, Kibbe WA, Lin SM. lumi: a pipeline for processing Illumina microarray. Bioinformatics. 2008;24(13):1547-8.

5. Phipson B, Lee S, Majewski IJ, Alexander WS, Smyth GK. Robust Hyperparameter Estimation Protects against Hypervariable Genes and Improves Power to Detect Differential Expression. Ann Appl Stat. 2016;10(2):946-63.

6. Ritchie ME, Phipson B, Wu D, Hu YF, Law CW, Shi W, et al. limma powers differential expression analyses for RNA-sequencing and microarray studies. Nucleic Acids Research. 2015;43(7).

7. Ritchie ME, Diyagama D, Neilson J, van Laar R, Dobrovic A, Holloway A, et al. Empirical array quality weights in the analysis of microarray data. BMC bioinformatics. 2006;7.

8. Benjamini Y, Hochberg Y. Controlling the False Discovery Rate: A Practical and Powerful Approach to Multiple Testing. Journal of the Royal Statistical Society Series B (Methodological). 1995;57(1):289-300.

9. Ntranos A, Casaccia P. Bromodomains: Translating the words of lysine acetylation into myelin injury and repair. Neurosci Lett. 2016;625:4-10.

10. Lang F, Shumilina E. Regulation of ion channels by the serum- and glucocorticoid-inducible kinase SGK1. FASEB J. 2013;27(1):3-12.

11. Baban B, Liu JY, Mozaffari MS. SGK-1 regulates inflammation and cell death in the ischemic-reperfused heart: pressure-related effects. Am J Hypertens. 2014;27(6):846-56.
